# Survival Benefit of Adjuvant Chemotherapy After Pancreatoduodenectomy for Ampullary Adenocarcinoma: a Propensity-Matched National Cancer Database (NCDB) Analysis

**DOI:** 10.1007/s11605-020-04879-x

**Published:** 2020-11-23

**Authors:** Sivesh K. Kamarajah, Filip Bednar, Clifford S. Cho, Hari Nathan

**Affiliations:** 1 Department of Surgery, University Hospital Birmingham NHS Trust, Birmingham, UK; 2 Department of Hepatobiliary, Pancreatic and Transplant Surgery, Freeman Hospital, Newcastle Upon Tyne, Tyne and Wear UK; 3 Department of Surgery, Freeman Hospital, Newcastle Upon Tyne, Tyne and Wear UK; 4 Department of Surgery, 2210A Taubman Health Care Center, University of Michigan, 1500 E Medical Center Dr, SPC 5343, Ann Arbor, MI 48109-5343 USA

**Keywords:** Ampullary cancer, Adjuvant therapy, Resection

## Abstract

**Background:**

The benefit of adjuvant chemotherapy (AC) after pancreatoduodenectomy (PD) for ampullary adenocarcinoma is uncertain. We aimed to evaluate the association of AC with survival in patients with resected ampullary adenocarcinoma.

**Methods:**

Using the National Cancer Database (NCDB) data from 2004 to 2016, patients with non-metastatic ampullary adenocarcinoma who underwent PD were identified. Patients with neoadjuvant radiotherapy and chemotherapy and survival < 6 months were excluded. Propensity score matching was used to account for treatment selection bias. A multivariable Cox proportional hazards model was then used to analyze the association of AC with survival.

**Results:**

Of 3186 (43%) AC and 4172 (57%) no AC (noAC) patients, 1720 AC and 1720 noAC patients remained in the cohort after matching. Clinicopathologic variables were well balanced after matching. After matching, AC was associated with improved survival (median 47.5 vs 39.6 months, *p* = 0.003), which remained after multivariable adjustment (HR: 0.83, CI_95%_: 0.76–0.91, *p* < 0.001). Multivariable interaction analyses showed that this benefit was seen irrespective of nodal status: N0 (HR: 0.81, CI_95%_: 0.68–0.97, *p* < 0.001), N1 (HR: 0.65, CI_95%_: 0.61–0.70, *p* < 0.001), N2 (HR: 0.73, CI_95%_: 0.59–0.90, *p* = 0.003), N3 (HR: 0.59, CI_95%_: 0.44–0.78, *p* < 0.001); and margin status: R0 (HR: 0.85, CI_95%_: 0.77–0.94, *p* < 0.001), R1 (HR: 0.69, CI_95%_: 0.48–1.00, *p* < 0.001). Stratified analyses by nodal and margin status demonstrated consistent results.

**Conclusion:**

In this large retrospective cohort study, AC after resected ampullary adenocarcinoma was associated with a survival benefit in patients, including patients with node-negative and margin-negative disease.

**Supplementary Information:**

The online version contains supplementary material available at 10.1007/s11605-020-04879-x.

## Introduction

Ampullary adenocarcinoma typically has a better long-term prognosis after curative resection than other periampullary cancers, with 5-year survival rates ranging from 30 to 70%.^1^–^5^ Despite this, up to 50% of patients have recurrence,^6^,^7^ with some series^8^,^9^ demonstrating similar rates of locoregional and distant recurrence while others^10^,^11^ suggesting predominance of distant recurrence. Adjuvant chemotherapy (AC) may help reduce both locoregional and distant recurrence rates and improve overall survival. While multiple randomized controlled trials have conclusively established the survival benefit of AC for pancreatic cancer,^12^–^17^ its role is not yet clear for ampullary adenocarcinoma.

High-quality evidence on AC for periampullary adenocarcinoma is lacking. First, randomized controlled trials (RCTs)^18^–^21^ and meta-analyses^22^,^23^ have demonstrated no survival benefit. However, different periampullary cancers (i.e., distal cholangiocarcinoma, duodenal adenocarcinoma, ampullary adenocarcinoma, and pancreatic adenocarcinoma) have varying prognoses, genetic profiles^24^, and likely responses to AC.^25^ Because ampullary adenocarcinoma is relatively uncommon, recruitment to RCTs has only been possible together with other periampullary cancer, and no RCT focused on ampullary adenocarcinoma exists. Subgroup analyses of these RCTs have limited interpretability and are prone to type II error. Retrospective single-center, multi-institutional series offer conflicting evidence regarding the benefit of AC.^2^,^10^,^25^–^31^ Therefore, the use of AC after pancreatoduodenectomy (PD) for ampullary adenocarcinoma remains controversial, especially in patients thought to be at a lower risk for recurrence, such as those with margin-negative resections and node-negative disease.

We sought to add evidence to this debate by performing a large, nationwide, high-quality retrospective study to assess the potential benefit of AC after PD for ampullary adenocarcinoma. With contemporary data from the National Cancer Data Base (NCDB), the association of AC with survival after PD for ampullary adenocarcinoma was analyzed. Propensity-matched analysis was used to address treatment selection bias, and overall survival in clinically relevant subgroups of patients based on nodal and margin status was assessed.

## Methods

### Data Source

The NCDB is a joint project of the Commission on Cancer (CoC) of the American College of Surgeons and the American Cancer Society.^32^,^33^ The NCDB gathers information from approximately 1500 CoC-accredited hospitals and includes > 70% of all newly diagnosed malignancies in the USA. It contains specific details about patient demographics (age, sex, race, payer), facility type and location, tumor characteristics (size, grade, stage, histology), treatment course (type of surgery, receipt of chemotherapy, and radiation therapy), and outcomes (resection margins, lymph node status, and vital status).

### Study Population

The NCDB was used to identify all patients > 35 years old diagnosed with non-metastatic ampullary adenocarcinoma undergoing PD between 2004 and 2016. The International Classification of Disease for Oncology, Third Edition (ICD-O-3), classification was used to select adenocarcinoma histology (8140–8148) and excluded mucinous tumors, neuroendocrine tumors, and other histologies. Patients with other concomitant cancer diagnoses, those who received neoadjuvant chemotherapy or radiotherapy, and those with missing data on lymph node status and survival < 6 months were excluded.

The following patient-level characteristics were analyzed as provided by NCDB: age (36–50, 51–65, 66–80, > 80), race (white, black, other), Charlson-Deyo comorbidity score (CDCC), year of diagnosis, insurance status (Medicaid/Medicare, private insurance, uninsured), zip code–level education status (< 7%, 7–12.9%, 13–20.9%, ≥ 21%), zip code–level median household income (< $48,000, $48,000–$62,999, ≥ $63,000), and urban versus rural area of residence. The zip code–level education status represents the proportion of adults in the patient’s zip code who did not graduate from high school and is categorized as equally proportioned quartiles among all US zip codes. The following hospital-level characteristics were also analyzed: facility type (academic, community, other), facility location (Midwest, Northeast, South, West), and hospital distance from patient (< 12.5 miles, 12.5–49.9 miles, ≥ 50 miles). Finally, the following clinicopathologic characteristics were analyzed: nodal status (N0, N1, N2, N3), tumor grade/differentiation (well/moderate, poor/anaplastic, unknown), lymphovascular invasion (absent, present), and margin status (R0: negative; R1: positive).

Finally, the receipt of AC versus no adjuvant chemotherapy (noAC) as the primary exposure variable was analyzed. Coding for adjuvant therapy was derived using start of adjuvant therapy from diagnosis and surgery to obtain reliable estimates. However, discrimination between adjuvant radiotherapy-sensitizing chemotherapy was not possible based on the current available data.

### Statistical Analysis

Categorical variables were compared using the chi-square test. Non-normally distributed data were analyzed using the Mann-Whitney *U* test. Landmark analysis was also performed excluding early postoperative mortality (i.e., < 6 months) to account for immortal time bias.^34^ Survival was estimated using Kaplan-Meier survival curves and compared using the log-rank test. Multivariable analyses used Cox proportional hazards models. The conditional probability of receiving AC (i.e., the propensity score) was estimated using a multivariable logistic regression model including all patient- and hospital-level variables listed above. Next, balanced cohorts were created using 1-to-1 nearest-neighbor propensity score matching (PSM) without replacement (caliper width 0.1 standard deviations).^35^ Balance diagnostics were conducted by using standardized mean differences, with a value < 0.1 indicating good balance.^35^ The overall survival (OS) of matched patients with and without adjuvant chemotherapy was then evaluated. In order to address any residual confounding after PSM, multivariable Cox proportional hazards models again adjusted for all variables listed above, in addition to PSM. A stratified survival analysis by pathological node status (N0, N1, N2, and N3) and margin status (R0, R1) and interaction analyses between AC and pathological nodal and margin status were performed. A *p* value of < 0.05 was considered to be statistically significant. Data analysis was performed using the R Foundation Statistical software (R 3.2.2) with TableOne, ggplot2, Hmisc, Matchit, and survival packages (R Foundation for Statistical Computing, Vienna, Austria) as previously described.^36^ The study was deemed exempt from review by the University of Michigan Institutional Review Board.

## Results

### Patient Demographics and Clinicopathologic Characteristics

This study included 7358 patients with resected ampullary adenocarcinoma. Of these patients, 3186 (43%) received AC and 4172 (57%) did not. Median follow-up was 28 months (interquartile range 13–54 months). Baseline demographics of the unmatched cohort revealed that patients receiving AC were from high hospital volume and younger and had lower comorbidity burden (Table 1). There was a wide variation in receipt of AC by institution ranging from 0 to 100% (Supplementary Figure 1). Patients receiving AC also had larger, more locally invasive tumors and more positive lymph nodes, consistent with treatment selection bias. Patients receiving AC had significantly higher rates of lymph nodes examined compared to noAC (median: 18 vs 14, *p* < 0.001). Patients with node-positive disease were much more likely to receive AC than those with node-negative disease (72% vs 40%, *p* < 0.001). Patients with margin-positive disease were much more likely to receive AC than those with margin-negative disease (7% vs 4%, *p* < 0.001). Logistic regression identified advanced tumor, nodal involvement, and lymphovascular invasion as independent predictors of receipt of chemotherapy (Supplementary Table 1). To account for this treatment selection bias, PSM was performed as described above. This resulted in well-balanced cohorts (Table 1). Standardized mean differences were calculated for each variable and ranged between 0.01 and 0.05, indicating good balance.

**Table 1:** Clinicopathologic characteristics of ampullary adenocarcinoma by receipt of adjuvant chemotherapy in unmatched and matched cohort

		Unmatched cohort			Matched cohort		
		noAC *n* = 4172	AC *n* = 3186	*p* value	noAC *n* = 1720	AC *n* = 1720	*p* value
Hospital factors							
Center volume	1 (lowest)	508 (12.2)	573 (18.0)	< 0.001	261 (15.2)	261 (15.2)	0.998
	2	774 (18.6)	648 (20.3)		325 (18.9)	327 (19.0)	
	3	854 (20.5)	601 (18.9)		328 (19.1)	328 (19.1)	
	4	968 (23.2)	673 (21.1)		383 (22.3)	389 (22.6)	
	5 (highest)	1068 (25.6)	691 (21.7)		423 (24.6)	415 (24.1)	
Facility type	Community	1208 (29.0)	1038 (32.6)	< 0.001	954 (55.5)	953 (55.4)	0.933
	Academic	2451 (58.7)	1665 (52.3)		535 (31.1)	529 (30.8)	
	Others	513 (12.3)	483 (15.2)		231 (13.4)	238 (13.8)	
Facility location	Northeast	889 (21.3)	714 (22.4)	0.166	409 (23.8)	411 (23.9)	0.479
	South	1580 (37.9)	1150 (36.1)		358 (20.8)	390 (22.7)	
	Midwest	1031 (24.7)	764 (24.0)		646 (37.6)	609 (35.4)	
	West	672 (16.1)	558 (17.5)		307 (17.8)	310 (18.0)	
Patient factors							
Year of diagnosis	2006–2007	1646 (39.5)	431 (13.5)	< 0.001	385 (22.4)	375 (21.8)	0.339
	2008–2009	585 (14.0)	545 (17.1)		249 (14.5)	235 (13.7)	
	2010–2011	647 (15.5)	608 (19.1)		295 (17.2)	269 (15.6)	
	2012–2013	696 (16.7)	766 (24.0)		402 (23.4)	404 (23.5)	
	2014–2016	598 (14.3)	836 (26.2)		389 (22.6)	437 (25.4)	
Age at diagnosis (years)	36–50	272 (6.5)	384 (12.1)	< 0.001	156 (9.1)	179 (10.4)	0.420
	51–65	1339 (32.1)	1374 (43.1)		661 (38.4)	672 (39.1)	
	66–80	1998 (47.9)	1288 (40.4)		789 (45.9)	751 (43.7)	
	≥ 80	560 (13.4)	134 (4.2)		112 (6.5)	113 (6.6)	
Sex	Male	2334 (55.9)	1840 (57.8)	0.127	749 (43.5)	729 (42.4)	0.513
	Female	1838 (44.1)	1346 (42.2)		971 (56.5)	991 (57.6)	
CDCC score	0–1	3901 (93.5)	3010 (94.5)	0.093	1617 (94.0)	1619 (94.1)	0.942
	≥ 2	271 (6.5)	176 (5.5)		103 (6.0)	101 (5.9)	
Insurance status	Uninsured	266 (6.4)	188 (5.9)	< 0.001	92 (5.3)	102 (5.9)	0.500
	Private insurance	1323 (31.7)	1400 (43.9)		856 (49.8)	827 (48.1)	
	Medicaid	198 (4.7)	199 (6.2)		652 (37.9)	683 (39.7)	
	Medicare	2385 (57.2)	1399 (43.9)		120 (7.0)	108 (6.3)	
Education level	≥ 21%	782 (18.7)	535 (16.8)	< 0.001	433 (25.2)	426 (24.8)	0.976
	13–20.9%	1085 (26.0)	738 (23.2)		299 (17.4)	299 (17.4)	
	7–12.9%	1337 (32.0)	1074 (33.7)		415 (24.1)	410 (23.8)	
	< 7%	968 (23.2)	839 (26.3)		573 (33.3)	585 (34.0)	
Median income	≤ $47,999	1738 (41.7)	1141 (35.8)	< 0.001	636 (37.0)	622 (36.2)	0.808
	$48,000–$62,999	1128 (27.0)	902 (28.3)		470 (27.3)	486 (28.3)	
	≥ $63,000	1306 (31.3)	1143 (35.9)		614 (35.7)	612 (35.6)	
Tumor factors							
Tumor grade	Well	566 (13.6)	266 (8.3)	< 0.001	88 (5.1)	87 (5.1)	0.696
	Moderate	2232 (53.5)	1632 (51.2)		897 (52.2)	872 (50.7)	
	Poor	1125 (27.0)	1125 (35.3)		568 (33.0)	601 (34.9)	
	Anaplastic	249 (6.0)	163 (5.1)		167 (9.7)	160 (9.3)	
AJCC pathological T classification	T1	854 (20.5)	193 (6.1)	< 0.001	153 (8.9)	151 (8.8)	0.810
	T2	1400 (33.6)	876 (27.5)		534 (31.0)	509 (29.6)	
	T3	1132 (27.1)	1178 (37.0)		580 (33.7)	594 (34.5)	
	T4	786 (18.8)	939 (29.5)		453 (26.3)	466 (27.1)	
AJCC pathological N classification	N0	2574 (61.7)	896 (28.1)	< 0.001	679 (39.5)	644 (37.4)	0.389
	N1	1117 (26.8)	1469 (46.1)		697 (40.5)	695 (40.4)	
	N2	310 (7.4)	491 (15.4)		224 (13.0)	242 (14.1)	
	N3	171 (4.1)	330 (10.4)		120 (7.0)	139 (8.1)	
Margin status	Negative	4021 (96.4)	2968 (93.2)	< 0.001	1640 (95.3)	1628 (94.7)	0.389
	Positive	151 (3.6)	218 (6.8)		80 (4.7)	92 (5.3)	
Lymphovascular invasion	Absent	3584 (85.9)	2071 (65.0)	< 0.001	1257 (73.1)	1212 (70.5)	0.096
	Present	588 (14.1)	1115 (35.0)		463 (26.9)	508 (29.5)	
Treatment factors							
Adjuvant radiotherapy	No	3826 (91.7)	1609 (50.5)	< 0.001	1401 (81.5)	1396 (81.2)	0.861
	Yes	346 (8.3)	1577 (49.5)		319 (18.5)	324 (18.8)	

*Additional variables included into the propensity matching omitted from tables were hospital factors (hospital distance), patient factors (race, residence), and tumor factors (lymph nodes examined)*

*AC* adjuvant chemotherapy, *AJCC* American Joint Commission on Cancer, *CDCC* Charlson-Deyo comorbidity, *noAC* no adjuvant chemotherapy

### Association of Adjuvant Chemotherapy with Survival

**Fig. 1:**
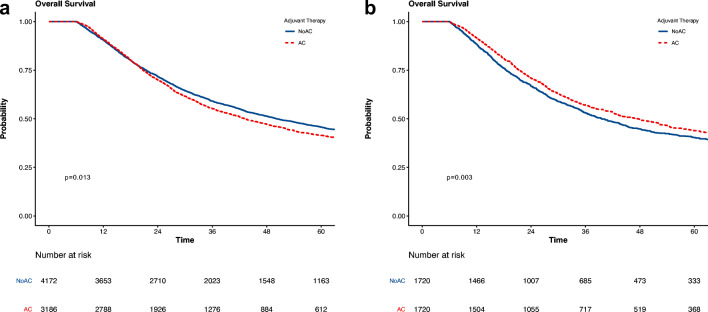
Overall survival of adjuvant chemotherapy following resection for ampullary adenocarcinoma in **a** unmatched and **b** matched cohorts

For the overall cohort, median survival was 40.2 months, and 5-year survival was 40%. In the unmatched cohort, the survival of patients receiving AC was significantly shorter than those who did not (median: 43.3 vs 50.2 months, 5-year 42% vs 46%, *p* = 0.013) (Fig. 1a, Table 2, and Supplementary Table 2). In the matched cohort, patients receiving AC still had a significant survival advantage (median 47.5 vs 39.6 months, 5-year 44% vs 40%, *p* < 0.001) (Fig. 1b and Table 2). In the PSM multivariable analysis, factors associated with adverse survival included older age, higher comorbidity score, advanced tumors, node-positive tumors, positive margin status, and lymphovascular invasion (Table 3). Patients receiving AC had improved survival after PSM and multivariable adjustment (HR: 0.83, CI_95%_: 0.76–0.91, *p* < 0.001) (Tables 2 and 3).

**Table 2:** Association of adjuvant chemotherapy with overall survival of patients with resected ampullary adenocarcinoma in unmatched and matched cohorts and stratified by nodal status and margin status for matched cohorts from multivariable Cox regression model

Cohort	Chemotherapy	Median survival (IQR), months	Hazard ratio (CI_95%_)	*p*-value
All patients				
Unmatched	noAC	50.2 (47.5–54.1)	REF	< 0.001
	AC	43.3 (40.6–46.5)	0.85 (0.78–0.92)	
Matched	noAC	39.6 (36.6–43.7)	REF	< 0.001
	AC	47.5 (42.5–52.3)	0.83 (0.76–0.91)	
Stratified by nodal status in matched cohort				
N0	noAC	86.1 (78.5–104.0)	REF	0.017
	AC	90.0 (74.9–NR)	0.80 (0.66–0.96)	
N1	noAC	34.4 (29.7–37.3)	REF	0.014
	AC	39.1 (34.4–44.8)	0.84 (0.73–0.97)	
N2	noAC	23.8 (21.4–29.7)	REF	0.014
	AC	27.3 (23.9–30.3)	0.76 (0.61–0.94)	
N3	noAC	21.0 (16.6–24.3)	REF	0.011
	AC	26.1 (21.4–31.5)	0.64 (0.46–0.90)	
Stratified by margin status in matched cohort				
R0	noAC	42.3 (38.3–45.5)	REF	0.001
	AC	49.2 (43.8–54.4)	0.85 (0.77–0.94)	
R1	noAC	17.5 (14.5–23.9)	REF	0.028
	AC	22.3 (19.7–28.1)	0.61 (0.39–0.95)	
Stratified by adjuvant radiotherapy status in matched cohort				
No adjuvant radiotherapy	noAC	42.0 (37.1–46.2)	REF	0.008
	AC	44.8 (41.9–52.3)	0.86 (0.78–0.96)	
Adjuvant radiotherapy	noAC	36.8 (30.1–40.9)	REF	< 0.001
	AC	51.1 (40.2–67)	0.68 (0.55–0.84)	

*AC* adjuvant chemotherapy, *CI* confidence interval, *IQR* interquartile range, *noAC* no adjuvant chemotherapy, *REF* referent

**Table 3:** Multivariable cox regression model of survival of patients with resected ampullary adenocarcinoma in the matched cohort

		Hazard ratio (CI_95%_)	*p* value
Hospital factors			
Center volume	1 (lowest)	REF	< 0.001
	2	0.84 (0.71–0.98)	
	3	0.94 (0.79–1.11)	
	4	0.88 (0.74–1.04)	
	5 (highest)	0.82 (0.68–0.98)	
Facility type	Community	REF	0.8
	Academic	0.97 (0.85–1.10)	
	Others	1.11 (0.95–1.28)	
Facility location	Northeast	REF	< 0.001
	South	0.83 (0.72–0.96)	
	Midwest	1.05 (0.93–1.20)	
	West	1.07 (0.92–1.24)	
Patient factors			
Year of diagnosis	2006–2007	REF	< 0.001
	2008–2009	1.01 (0.86–1.19)	
	2010–2011	0.71 (0.60–0.86)	
	2012–2013	0.65 (0.55–0.78)	
	2014–2016	0.68 (0.56–0.83)	
Age at diagnosis (years)	36–50	REF	< 0.001
	51–65	1.48 (1.22–1.79)	
	66–80	1.66 (1.34–2.05)	
	≥ 80	2.54 (1.95–3.29)	
	Missing	0.94 (0.23–3.88)	
Sex	Male	REF	< 0.001
	Female	1.12 (1.02–1.23)	
CDCC score	0–1	REF	< 0.001
	≥ 2	1.16 (0.97–1.40)	
Insurance status	Uninsured	REF	< 0.001
	Private insurance	0.79 (0.62–1.00)	
	Medicaid	0.73 (0.58–0.91)	
	Medicare	0.90 (0.68–1.18)	
Education level	≥ 21%	REF	0.02
	13–20.9%	0.83 (0.69–1.01)	
	7–12.9%	0.95 (0.81–1.12)	
	< 7%	1.03 (0.90–1.18)	
Median income	≤ $47,999	REF	< 0.001
	$48,000–$62,999	0.83 (0.73–0.95)	
	≥ $63,000	0.84 (0.72–0.98)	
Tumor factors			
Tumor grade	Well	REF	< 0.001
	Moderate	0.84 (0.69–1.03)	
	Poor	0.98 (0.80–1.21)	
	Anaplastic	0.67 (0.52–0.87)	
AJCC pathological T classification	T1	REF	< 0.001
	T2	0.95 (0.77–1.17)	
	T3	1.83 (1.51–2.23)	
	T4	1.72 (1.40–2.10)	
AJCC pathological N stage	N0	REF	< 0.001
	N1	1.61 (1.43–1.81)	
	N2	2.34 (2.01–2.71)	
	N3	2.31 (1.92–2.78)	
Margin status	Negative	REF	< 0.001
	Positive	1.65 (1.37–2.00)	
Lymphovascular invasion	Absent	REF	0.073
	Present	1.35 (1.18–1.55)	
Treatment factors			
Adjuvant radiotherapy	No	REF	< 0.001
	Yes	0.87 (0.75–1.01)	
Adjuvant chemotherapy	No	REF	< 0.001
	Yes	0.83 (0.76–0.91)	

*Additional variables included into the propensity matching omitted from tables were hospital factors (hospital distance), patient factors (race, residence), and tumor factors (lymph nodes examined)*

*AJCC* American Joint Commission on Cancer, *CDCC* Charlson-Deyo comorbidity, *CI* confidence interval, *REF* referent

### Interaction Between Adjuvant Chemotherapy and Nodal Status

**Fig. 2:**
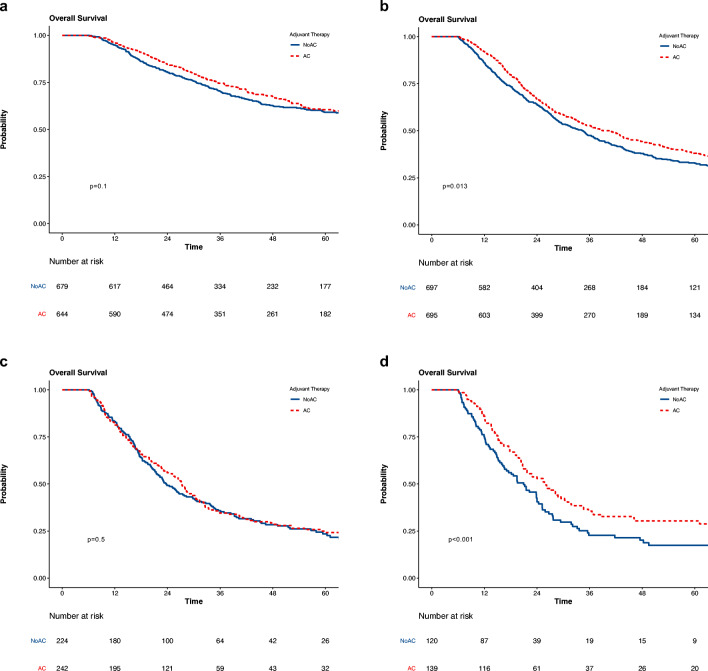
Overall survival of adjuvant chemotherapy following resection for ampullary adenocarcinoma stratified by nodal status in matched cohorts: **a** N0, **b** N1, **c** N2, **d** N3

Interaction analyses were performed to further understand the impact of AC by nodal status. In unadjusted analysis, there were significant differences in survival between AC and noAC patients in patients with N1 disease (median 39.1 vs 34.4 months, *p* = 0.014) (Fig. 2a) and N3 disease (median 26.1 vs 21.0 months, *p* = 0.011) (Fig. 2b) but not N0 disease (median 90.0 vs 86.1 months, *p* = 0.1) (Supplementary Figure 2A) and N2 disease (median 27.3 vs 23.8 months, *p* = 0.5) (Supplementary Figure 2B). In multivariable analyses modeling the interaction between receipt of AC and nodal status, a survival benefit again was seen for patients with N0, N1, N2, and N3 disease (Table 4 and Supplementary Table 3). As a sensitivity analysis, four separate multivariable analyses in cohorts including only those with N0, N1, N2, and N3 disease were performed, respectively. These analyses confirmed the same findings (Table 2).

**Table 4:** Multivariable cox regression model of survival of patients with resected ampullary adenocarcinoma in matched cohort, with interactions between chemotherapy and nodal status and margin status

		Hazard ratio (CI_95%_)	*p* value
Interaction by nodal status			
Adjuvant chemotherapy × AJCC pathological N stage	N0 + noAC	REF	0.001
	N0 + AC	0.81 (0.68–0.97)	
	N1 + noAC	1.60 (1.37–1.88)	
	N1 + AC	0.65 (0.61–0.70)	
	N2 + noAC	2.15 (1.75–2.64)	
	N2 + AC	0.73 (0.59–0.90)	
	N3 + noAC	2.51 (1.95–3.24)	
	N3 + AC	0.59 (0.44–0.78)	
Interaction by margin status			
Adjuvant chemotherapy × margin status	R0 + noAC	REF	< 0.001
	R0 + AC	0.85 (0.77–0.94)	
	R1 + noAC	2.00 (1.55–2.59)	
	R1 + AC	0.69 (0.48–1.00)	

*AC* adjuvant chemotherapy, *CDCC* Charlson-Deyo comorbidity, *CI* confidence interval, *noAC* no adjuvant chemotherapy, *REF* referent

### Interaction Between Adjuvant Chemotherapy and Margin Status

**Fig. 3:**
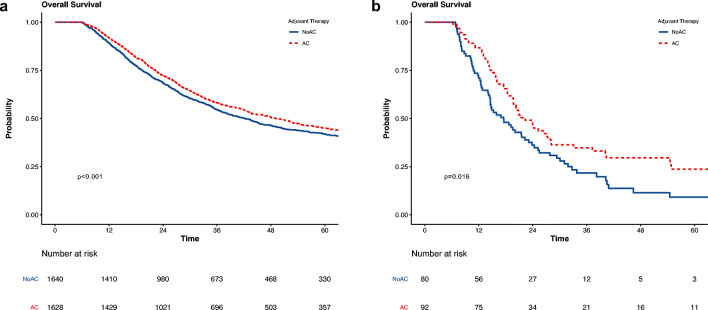
Overall survival of adjuvant chemotherapy following resection for ampullary adenocarcinoma stratified by margin status in matched cohorts: **a** R0, **b** R1

Interaction analyses were performed to further understand the impact of AC by margin status. In unadjusted analysis, there were significant differences in survival between AC and noAC patients in patients with R0 disease (median 49.2 vs 42.3 months, *p* < 0.001) (Fig. 3a) and in patients with R1 disease (median 22.3 vs 17.5 months, *p* = 0.016) (Fig. 3b). In multivariable analyses modeling the interaction between receipt of AC and margin status, a survival benefit again was seen for patients with R0 (HR: 0.85, CI_95%_: 0.77–0.94, *p* < 0.001) and R1 margin status (HR: 0.69, CI_95%_: 0.48–1.00, *p* < 0.001) (Table 4 and Supplementary Table 4). As a sensitivity analysis, we performed two separate multivariable analyses in cohorts including only those with R0 or R1 margin, respectively. These analyses confirmed the same findings (Table 2).

### Association of Adjuvant Chemotherapy and Radiotherapy with Survival

Additional analyses were performed to further understand the impact of AC in the setting of adjuvant radiotherapy. In unadjusted analysis, there were no significant differences in survival between AC and noAC patients in patients without adjuvant radiotherapy (median 44.8 vs 42.0 months, *p* = 0.2) (Supplementary Figure 3A), but significantly longer with AC than noAC in patients with adjuvant radiotherapy (median 51.1 vs 36.8 months, *p* < 0.001) (Supplementary Figure 3B). In multivariable analyses modeling the interaction between receipt of AC and radiotherapy, a survival benefit again was seen for patients without adjuvant radiotherapy (HR: 0.81, CI_95%_: 0.68–0.96, *p* < 0.001) and with adjuvant radiotherapy (HR: 0.56, CI_95%_: 0.47–0.66, *p* < 0.001) (Supplementary Table 5). As a sensitivity analysis, we performed two separate multivariable analyses in cohorts including only those without and with adjuvant radiotherapy, respectively. These analyses confirmed the same findings (Table 2).

## Discussion

Ampullary adenocarcinoma remains a relatively uncommon malignancy without broadly accepted protocols for optimal multimodality management following curative-intent resection. As such, there remains an ongoing dilemma regarding the role of AC after PD for ampullary adenocarcinoma, and practice varies significantly. In this large national registry analysis including 8307 patients, AC after resected ampullary adenocarcinoma was associated with improved survival after multivariable adjustment and accounting for treatment selection bias. Stratified analyses revealed that this benefit was maintained irrespective of pathological nodal involvement and resection margin status. Sensitivity landmark analyses excluding early postoperative deaths also demonstrated consistent findings favoring AC.^34^ As such, these data suggest a benefit to routine use of AC for ampullary adenocarcinoma, even in the absence of nodal involvement or compromised surgical margins. Broad acceptance of the routine use of AC for ampullary adenocarcinoma should be considered in the multimodality treatment of ampullary adenocarcinoma, just as in pancreatic cancer.

Current evidence for AC in resected ampullary adenocarcinoma is limited to retrospective case series. Recent institutional series by Ecker et al.^26^ (*n* = 357 patients; HR: 0.90; CI_95%_: 0.51–1.56), Bolm et al.^30^ with 214 patients (median: 85.0 vs 65.0 months), and Moekotte et al.^31^ with 1,163 patients (median: not reached vs 32 months) demonstrated no survival benefit with AC. However, these studies are limited by small institutional cohorts and selection bias. Subgroup analyses of the landmark ESPAC-3^20^ RCT in patients with ampullary adenocarcinoma (*n* = 297 patients) demonstrated no statistically significant differences in survival between patients receiving gemcitabine, 5-fluorouracil, and no chemotherapy (median: 70.8 vs 57.8 vs 40.6 months). This is possibly a result of a type II error. The only level I evidence on the role of AC in clinical practice is drawn from subgroup analyses of RCTs 18,20,21 in periampullary cancers, which have their own limitations. This large study, while still retrospective, used robust methods to account for treatment selection bias and still demonstrated survival benefit with AC.

The presence of high-risk factors, such as nodal involvement or positive margins, is commonly used to select patients for adjuvant therapy, as evidence by the distribution AC use in the unmatched cohort. To the authors’ knowledge, no published studies have explored the role of AC specifically in patients with node-negative disease or negative margins. Such treatment decisions likely reflect an estimation of the risk of systemic recurrence, which clearly is lower in patients with node-negative, margin-negative resections. However, systemic recurrence in such patients may still be as high as 40% 8,37, and local recurrence as high as 50%.^38^, ^39^ Our results suggest that AC has a role in these subgroups of patients by reducing or delaying recurrence and prolonging survival. Nevertheless, there may be a more select subgroup of patients in whom the benefit of AC does not outweigh the risk^40^, especially those with intestinal-type rather than pancreaticobiliary-type tumors, given their more favorable overall prognosis.^41^ Unfortunately, NCDB data do not allow these subtypes to be distinguished. However, if intestinal-type tumors have no true benefit or less benefit from adjuvant chemotherapy, the implication from the survival data is that pancreaticobiliary-type tumors have even more benefit than estimated.

Several limitations of our study should be acknowledged. First, despite the use of PSM to address treatment selection bias, the potential for residual bias remains in this retrospective cohort study. Second, the duration of adjuvant chemotherapy and the specific regimens used are not available from NCDB. Third, this study did not assess the role of neoadjuvant RT, which may or may not be associated with a similar survival benefit. Fourth, pathologic assessment of tumors in the periampullary region can be challenging, as the site or origin (true ampullary vs other peri-ampullary) may be difficult to ascertain for larger tumors. However, this limitation applies to any study that uses histopathologic analysis for diagnosis. Fifth, patients with survival of < 6 months were excluded as it is likely that these patient cohorts may not have completed course of adjuvant chemotherapy due to death. However, it is unclear if these patients had complications related to adjuvant therapy. Finally, because NCDB does not include data on recurrence patterns or disease-free survival, we can only speculate as to whether improved survival was associated with local or systemic disease control.

## Conclusion

In this large nationwide retrospective study, AC was associated with a survival benefit in patients with resected ampullary adenocarcinoma, regardless of pathological nodal involvement, resection margin status, and receipt of adjuvant radiotherapy. These data suggest AC should be broadly considered in the multimodality treatment of ampullary adenocarcinoma.

## Supplementary Information

ESM 1 (DOCX 1776 kb)
