# Supplementary material for: Survival Benefit of Adjuvant Chemotherapy After Pancreatoduodenectomy for Ampullary Adenocarcinoma: a Propensity-Matched National Cancer Database (NCDB) Analysis
Source: J Gastrointest Surg. 2020 Nov 23;25(7):1805–14. doi: 10.1007/s11605-020-04879-x (PMC8275534; doi:10.1007/s11605-020-04879-x)
Supplement: Supplementary file 1 — (DOCX 1776 kb) [file 11605_2020_4879_MOESM1_ESM.docx]

**Survival Benefit of Adjuvant Chemotherapy after Pancreatoduodenectomy for Ampullary Adenocarcinoma: A Propensity-Matched National Cancer Database (NCDB) Analysis**

Sivesh K Kamarajah, BMedSci MBChB,^1^ Filip Bednar, MD PhD,^2^ Clifford S. Cho, MD,^2^ Hari Nathan, MD PhD FACS^2^

1. Department of Surgery, University Hospital Birmingham NHS Trust, Birmingham, United Kingdom
2. Department of Surgery, University of Michigan, Ann Arbor, MI, USA

**Running title**: Adjuvant chemotherapy for ampullary cancers

**Corresponding Author**:

Hari Nathan, MD, PhD, FACS

Assistant Professor

Department of Surgery

University of Michigan

2210A Taubman Health Care Center

1500 E Medical Center Dr, SPC 5343

Ann Arbor, MI 48109-5343

e-mail: [drnathan@umich.edu](mailto:drnathan@umich.edu)

**Manuscript Type:** Original Article

**Number of Text Pages**: 12

**Number of Tables**: 4

**Number of Figures**: 3

**Word Count**: 2,473

**Funding source**: None declared

**Conflict of interest**: None declared

# Supplementary Table 1 Logistic regression associated with receipt of adjuvant chemotherapy for ampullary adenocarcinoma in unmatched cohort

|  |  | **Odds ratio (CI_95%_)** | **p-value** |
| --- | --- | --- | --- |
| **Hospital Factors** |  |  |  |
| Center Volume | 1 (Lowest) | REF | 0.002 |
|  | 2 | 0.81 (0.65-1.00) |  |
|  | 3 | 0.72 (0.58-0.90) |  |
|  | 4 | 0.78 (0.62-0.99) |  |
|  | 5 (Highest) | 0.80 (0.62-1.02) |  |
| Facility Type | Community | REF | 0.9 |
|  | Academic | 0.99 (0.83-1.17) |  |
|  | Others | 0.96 (0.78-1.19) |  |
| Facility Location | Northeast | REF | 0.6 |
|  | South | 0.95 (0.80-1.12) |  |
|  | Midwest | 0.92 (0.76-1.10) |  |
|  | West | 0.96 (0.79-1.18) |  |
| **Patient Factors** |  |  |  |
| Year of Diagnosis | 2006-2007 | REF | <0.001 |
|  | 2008-2009 | 7.62 (6.13-9.49) |  |
|  | 2010-2011 | 6.86 (5.50-8.58) |  |
|  | 2012-2013 | 9.60 (7.73-11.98) |  |
|  | 2014-2016 | 13.17 (10.58-16.47) |  |
| Age at Diagnosis, years | 36-50 | REF | <0.001 |
|  | 51-65 | 0.69 (0.55-0.87) |  |
|  | 66-80 | 0.51 (0.40-0.66) |  |
|  | ≥80 | 0.20 (0.14-0.27) |  |
| Sex | Male | REF | 0.2 |
|  | Female | 0.99 (0.88-1.12) |  |
| CDCC Score | 0-1 | REF | 0.010 |
|  | ≥2 | 0.79 (0.61-1.02) |  |
| Insurance Status | Uninsured | REF | 0.002 |
|  | Private Insurance | 1.46 (1.12-1.91) |  |
|  | Medicaid | 1.22 (0.85-1.74) |  |
|  | Medicare | 1.28 (0.97-1.70) |  |
| Education Level | ≥21% | REF | 0.4 |
|  | 13%-20.9% | 1.08 (0.89-1.31) |  |
|  | 7%-12.9% | 1.22 (1.00-1.50) |  |
|  | <7% | 1.21 (0.95-1.54) |  |
| Median Income | ≤$47,999 | REF | 0.3 |
|  | $48,000-$62,999 | 1.12 (0.95-1.33) |  |
|  | ≥$63,000 | 1.10 (0.90-1.34) |  |
| **Tumor Factors** |  |  |  |
| Tumor Grade | Well | REF | 0.023 |
|  | Moderate | 1.15 (0.94-1.41) |  |
|  | Poor | 1.36 (1.09-1.70) |  |
|  | Anaplastic | 1.39 (1.01-1.93) |  |
| AJCC Pathological T Classification | pT1 | REF | <0.001 |
|  | pT2 | 1.90 (1.53-2.36) |  |
|  | pT3 | 2.50 (2.02-3.12) |  |
|  | pT4 | 2.37 (1.88-3.00) |  |
| AJCC Pathological N Classification | N0 | REF | <0.001 |
|  | N1 | 2.50 (2.17-2.87) |  |
|  | N2 | 2.64 (2.14-3.25) |  |
|  | N3 | 3.03 (2.34-3.93) |  |
| Margin Status | Negative | REF | 0.4 |
|  | Positive | 1.17 (0.88-1.55) |  |
| Lymphovascular Invasion | Absent | REF | 0.003 |
|  | Present | 1.37 (1.16-1.61) |  |
| **Treatment Factors** |  |  |  |
| Adjuvant Radiotherapy | No | REF | <0.001 |
|  | Yes | 14.28 (12.03-17.05) |  |

**Abbreviations: CDCC: Charlson-Deyo comorbidity, CI: confidence interval **Additional variables included into the propensity matching omitted from tables were hospital factors (hospital distance), patient factors (race, residence) and tumor factors (lymph nodes examined).*

# Supplementary Table 2 Cox regression on overall survival for ampullary adenocarcinoma in unmatched cohort

|  | |  | | | **Hazard ratio (CI_95%)_** | | | **p-value** | | | | |
| --- | --- | --- | --- | --- | --- | --- | --- | --- | --- | --- | --- | --- |
| **Hospital Factors** | |  | | |  | | | | |  | |  |
| Center Volume | | 1 (Lowest) | | | REF | | | <0.001 | | | | |
|  | | 2 | | | 0.87 (0.78-0.97) | | |  | | | | |
|  | | 3 | | | 0.85 (0.76-0.95) | | |  | | | | |
|  | | 4 | | | 0.84 (0.75-0.95) | | |  | | | | |
|  | | 5 (Highest) | | | 0.77 (0.68-0.88) | | |  | | | | |
| Facility Type | | Community | | | REF | | | 0.8 | | | | |
|  | | Academic | | | 1.07 (0.98-1.17) | | |  | | | | |
|  | | Others | | | 1.13 (1.01-1.25) | | |  | | | | |
| Facility Location | | Northeast | | | REF | | | <0.001 | | | | |
|  | | South | | | 1.21 (1.10-1.32) | | |  | | | | |
|  | | Midwest | | | 1.10 (0.99-1.21) | | |  | | | | |
|  | | West | | | 1.23 (1.10-1.36) | | |  | | | | |
| **Patient Factors** | |  | | |  | | | | |  | |  |
| Year of Diagnosis | | 2006-2007 | | | REF | | | <0.001 | | | | |
|  | | 2008-2009 | | | 0.97 (0.88-1.06) | | |  | | | | |
|  | | 2010-2011 | | | 0.82 (0.74-0.91) | | |  | | | | |
|  | | 2012-2013 | | | 0.73 (0.65-0.81) | | |  | | | | |
|  | | 2014-2016 | | | 0.73 (0.64-0.83) | | |  | | | | |
| Age at Diagnosis, years | | 36-50 | | | REF | | | <0.001 | | | | |
|  | | 51-65 | | | 1.39 (1.22-1.59) | | |  | | | | |
|  | | 66-80 | | | 1.63 (1.41-1.89) | | |  | | | | |
|  | | ≥80 | | | 2.35 (1.99-2.79) | | |  | | | | |
| Sex | | Male | | | REF | | | <0.001 | | | | |
|  | | Female | | | 0.90 (0.85-0.96) | | |  | | | | |
| CDCC Score | | 0-1 | | | REF | | | <0.001 | | | | |
|  | | ≥2 | | | 1.21 (1.07-1.37) | | |  | | | | |
| Insurance Status | | Uninsured | | | REF | | | <0.001 | | | | |
|  | | Private Insurance | | | 0.91 (0.79-1.05) | | |  | | | | |
|  | | Medicaid | | | 1.19 (0.98-1.44) | | |  | | | | |
|  | | Medicare | | | 1.02 (0.88-1.19) | | |  | | | | |
| Education Level | | ≥21% | | | REF | | | 0.02 | | | | |
|  | | 13%-20.9% | | | 1.08 (0.98-1.20) | | |  | | | | |
|  | | 7%-12.9% | | | 1.13 (1.01-1.25) | | |  | | | | |
|  | | <7% | | | 1.08 (0.95-1.22) | | |  | | | | |
| Median Income | | ≤$47,999 | | | REF | | | <0.001 | | | | |
|  | | $48,000-$62,999 | | | 0.88 (0.80-0.95) | | |  | | | | |
|  | | ≥$63,000 | | | 0.81 (0.73-0.90) | | |  | | | | |
| **Tumor Factors** |  | |  |  | |  |  | |  | |  | |
| Tumor Grade | | Well | | | REF | | | <0.001 | | | | |
|  | | Moderate | | | 1.15 (1.03-1.29) | | |  | | | | |
|  | | Poor | | | 1.40 (1.24-1.58) | | |  | | | | |
|  | | Anaplastic | | | 1.36 (1.16-1.61) | | |  | | | | |
| AJCC Pathological T Classification | | T1 | | | REF | | | <0.001 | | | | |
|  | | T2 | | | 1.05 (0.93-1.18) | | |  | | | | |
|  | | T3 | | | 1.80 (1.61-2.02) | | |  | | | | |
|  | | T4 | | | 1.86 (1.65-2.10) | | |  | | | | |
| AJCC Pathological N Classification | | N0 | | | REF | | | <0.001 | | | | |
|  | | N1 | | | 1.69 (1.56-1.83) | | |  | | | | |
|  | | N2 | | | 2.27 (2.04-2.53) | | |  | | | | |
|  | | N3 | | | 2.75 (2.43-3.12) | | |  | | | | |
| Margin Status | | Negative | | | REF | | | <0.001 | | | | |
|  | | Positive | | | 1.65 (1.46-1.88) | | |  | | | | |
| Lymphovascular Invasion | | Absent | | | REF | | | 0.073 | | | | |
|  | | Present | | | 1.16 (1.05-1.28) | | |  | | | | |
| **Treatment Factors** | |  | | |  | | | | |  | |  |
| Adjuvant Radiotherapy | | No | | | REF | | | <0.001 | | | | |
|  | | Yes | | | 0.93 (0.86-1.01) | | |  | | | | |
| Adjuvant Chemotherapy | | No | | | REF | | | <0.001 | | | | |
|  | | Yes | | | 0.85 (0.78-0.92) | | |  | | | | |

*Abbreviations: AC: adjuvant chemotherapy, CDCC: Charlson-Deyo comorbidity, CI: confidence interval* ***Additional variables included into the propensity matching omitted from tables were hospital factors (hospital distance), patient factors (race, residence) and tumor factors (lymph nodes examined).*

# Supplementary Table 3 Multivariable cox regression model of survival of patients with resected ampullary adenocarcinoma in matched cohort, with interactions between adjuvant chemotherapy and nodal status

|  | |  | | | | | **Hazard ratio (CI_95%_)** | | | | | **p-value** | | | | |  |  |
| --- | --- | --- | --- | --- | --- | --- | --- | --- | --- | --- | --- | --- | --- | --- | --- | --- | --- | --- |
| **Hospital Factors** | |  | | | | |  | | | | | |  | | | |  |  |
| Center Volume | | 1 (Lowest) | | | | | REF | | | | | 0.002 | | | | |  |  |
|  | | 2 | | | | | 0.83 (0.71-0.98) | | | | |  | | | | |  |  |
|  | | 3 | | | | | 0.93 (0.79-1.10) | | | | |  | | | | |  |  |
|  | | 4 | | | | | 0.88 (0.74-1.04) | | | | |  | | | | |  |  |
|  | | 5 (Highest) | | | | | 0.81 (0.68-0.98) | | | | |  | | | | |  |  |
| Facility Type | | Community | | | | | REF | | | | | 0.8 | | | | |  |  |
|  | | Academic | | | | | 0.97 (0.85-1.10) | | | | |  | | | | |  |  |
|  | | Others | | | | | 1.11 (0.96-1.29) | | | | |  | | | | |  |  |
| Facility Location | | Northeast | | | | | REF | | | | | 0.001 | | | | |  |  |
|  | | South | | | | | 0.83 (0.72-0.96) | | | | |  | | | | |  |  |
|  | | Midwest | | | | | 1.05 (0.93-1.20) | | | | |  | | | | |  |  |
|  | | West | | | | | 1.07 (0.92-1.25) | | | | |  | | | | |  |  |
| **Patient Factors** | | |  | | | | |  | | | | | | |  | |  |  |
| Year of Diagnosis | | 2006-2007 | | | | | REF | | | | | 0.2 | | | | |  |  |
|  | | 2008-2009 | | | | | 1.01 (0.86-1.19) | | | | |  | | | | |  |  |
|  | | 2010-2011 | | | | | 0.71 (0.60-0.86) | | | | |  | | | | |  |  |
|  | | 2012-2013 | | | | | 0.65 (0.55-0.78) | | | | |  | | | | |  |  |
|  | | 2014-2016 | | | | | 0.67 (0.55-0.82) | | | | |  | | | | |  |  |
| Age at Diagnosis, years | | 36-50 | | | | | REF | | | | | <0.001 | | | | |  |  |
|  | | 51-65 | | | | | 1.48 (1.22-1.79) | | | | |  | | | | |  |  |
|  | | 66-80 | | | | | 1.66 (1.34-2.05) | | | | |  | | | | |  |  |
|  | | ≥80 | | | | | 2.56 (1.97-3.32) | | | | |  | | | | |  |  |
| Sex | | Male | | | | | REF | | | | | 0.002 | | | | |  |  |
|  | | Female | | | | | 1.13 (1.02-1.24) | | | | |  | | | | |  |  |
| CDCC Score | | 0-1 | | | | | REF | | | | | 0.001 | | | | |  |  |
|  | | ≥2 | | | | | 1.16 (0.97-1.39) | | | | |  | | | | |  |  |
| Insurance Status | | Uninsured | | | | | REF | | | | | 0.001 | | | | |  |  |
|  | | Private Insurance | | | | | 0.80 (0.63-1.01) | | | | |  | | | | |  |  |
|  | | Medicaid | | | | | 0.73 (0.58-0.92) | | | | |  | | | | |  |  |
|  | | Medicare | | | | | 0.90 (0.68-1.19) | | | | |  | | | | |  |  |
| Median Income | | ≤$47,999 | | | | | REF | | | | | 0.003 | | | | |  |  |
|  | | $48,000-$62,999 | | | | | 0.83 (0.73-0.95) | | | | |  | | | | |  |  |
|  | | ≥$63,000 | | | | | 0.84 (0.72-0.98) | | | | |  | | | | |  |  |
| **Tumor Factors** |  | | | |  |  | | | |  |  | | |  | | |  |  |
| Tumor Grade | | Well | | | | | REF | | | | | <0.001 | | | | |  |  |
|  | | Moderate | | | | | 0.86 (0.70-1.05) | | | | |  | | | | |  |  |
|  | | Poor | | | | | 0.99 (0.80-1.22) | | | | |  | | | | |  |  |
|  | | Anaplastic | | | | | 0.69 (0.53-0.89) | | | | |  | | | | |  |  |
| AJCC Pathological T Classification | | T1 | | | | | REF | | | | | <0.001 | | | | |  |  |
|  | | T2 | | | | | 0.95 (0.78-1.17) | | | | |  | | | | |  |  |
|  | | T3 | | | | | 1.84 (1.51-2.24) | | | | |  | | | | |  |  |
|  | | T4 | | | | | 1.72 (1.40-2.11) | | | | |  | | | | |  |  |
| Margin Status | | Negative | | | | | REF | | | | | <0.001 | | | | |  |  |
|  | | Positive | | | | | 1.66 (1.38-2.01) | | | | |  | | | | |  |  |
| Lymphovascular Invasion | | Absent | | | | | REF | | | | | <0.001 | | | | |  |  |
|  | | Present | | | | | 1.35 (1.17-1.54) | | | | |  | | | | |  |  |
| **Treatment Factors** | | | |  | | | | |  | | | | | | |  | | |
| Adjuvant Radiotherapy | | No | | | | | REF | | | | | <0.001 | | | | |  |  |
|  | | Yes | | | | | 0.83 (0.72-0.95)) | | | | |  | | | | |  |  |
| Adjuvant Chemotherapy * AJCC Pathological N Stage | | N0 + noAC | | | | | REF | | | | | 0.001 | | | | |  |  |
|  |  | N0 + AC | | | | | 0.81 (0.68-0.97) | | | | |  | | | | |  |  |
|  |  | N1 + noAC | | | | | 1.60 (1.37-1.88) | | | | |  | | | | |  |  |
|  |  | N1 + AC | | | | | 0.65 (0.61-0.70)) | | | | |  | | | | |  |  |
|  |  | N2 + noAC | | | | | 2.15 (1.75-2.64) | | | | |  | | | | |  |  |
|  |  | N2 + AC | | | | | 0.73 (0.59-0.90)) | | | | |  | | | | |  |  |
|  |  | N3 + noAC | | | | | 2.51 (1.95-3.24) | | | | |  | | | | |  |  |
|  |  | N3 + AC | | | | | 0.59 (0.44-0.78)) | | | | |  | | | | |  |  |

# *Abbreviations: AC: adjuvant chemotherapy, CDCC: Charlson-Deyo comorbidity, CI: confidence interval, REF: referent **Additional variables included into the propensity matching omitted from tables were hospital factors (hospital distance), patient factors (race, education level, residence) and tumor factors (lymph nodes examined).*

# Supplementary Table 4 Multivariable Cox regression model of survival of patients with resected ampullary adenocarcinoma in matched cohort, with interactions between chemotherapy and margin status

|  | |  | | | | | **Hazard ratio (CI_95%_)** | | | | | | | **p-value** | | | |  |  |  |  |
| --- | --- | --- | --- | --- | --- | --- | --- | --- | --- | --- | --- | --- | --- | --- | --- | --- | --- | --- | --- | --- | --- |
| **Hospital Factors** | |  | | | | |  | | | | |  | | | | | | |  |  |  |
| Center Volume | | 1 (Lowest) | | | | | REF | | | | | | | 0.002 | | | |  |  |  |  |
|  | | 2 | | | | | 0.84 (0.71-0.98) | | | | | | |  | | | |  |  |  |  |
|  | | 3 | | | | | 0.93 (0.79-1.10) | | | | | | |  | | | |  |  |  |  |
|  | | 4 | | | | | 0.88 (0.74-1.04) | | | | | | |  | | | |  |  |  |  |
|  | | 5 (Highest) | | | | | 0.81 (0.68-0.98) | | | | | | |  | | | |  |  |  |  |
| Facility Type | | Community | | | | | REF | | | | | | | 0.8 | | | |  |  |  |  |
|  | | Academic | | | | | 0.96 (0.84-1.10) | | | | | | |  | | | |  |  |  |  |
|  | | Others | | | | | 1.10 (0.95-1.27) | | | | | | |  | | | |  |  |  |  |
| Facility Location | | Northeast | | | | | REF | | | | | | | 0.001 | | | |  |  |  |  |
|  | | South | | | | | 0.83 (0.72-0.96) | | | | | | |  | | | |  |  |  |  |
|  | | Midwest | | | | | 1.06 (0.93-1.20) | | | | | | |  | | | |  |  |  |  |
|  | | West | | | | | 1.07 (0.92-1.25) | | | | | | |  | | | |  |  |  |  |
| **Patient Factors** | | |  | | | | |  | | | | |  | | | |  |  |  |  |  |
| Year of Diagnosis | | 2006-2007 | | | | | REF | | | | | | | 0.2 | | | |  |  |  |  |
|  | | 2008-2009 | | | | | 1.01 (0.86-1.19) | | | | | | |  | | | |  |  |  |  |
|  | | 2010-2011 | | | | | 0.72 (0.60-0.86) | | | | | | |  | | | |  |  |  |  |
|  | | 2012-2013 | | | | | 0.65 (0.54-0.78) | | | | | | |  | | | |  |  |  |  |
|  | | 2014-2016 | | | | | 0.68 (0.55-0.82) | | | | | | |  | | | |  |  |  |  |
| Age at Diagnosis, years | | 36-50 | | | | | REF | | | | | | | <0.001 | | | |  |  |  |  |
|  | | 51-65 | | | | | 1.48 (1.22-1.79) | | | | | | |  | | | |  |  |  |  |
|  | | 66-80 | | | | | 1.67 (1.35-2.06) | | | | | | |  | | | |  |  |  |  |
|  | | ≥80 | | | | | 2.56 (1.97-3.32) | | | | | | |  | | | |  |  |  |  |
|  | | Missing | | | | | 0.94 (0.23-3.86) | | | | | | |  | | | |  |  |  |  |
| Sex | | Male | | | | | REF | | | | | | | 0.002 | | | |  |  |  |  |
|  | | Female | | | | | 0.84 (0.77 - 0.92) | | | | | | |  | | | |  |  |  |  |
| CDCC Score | | 0-1 | | | | | REF | | | | | | | 0.001 | | | |  |  |  |  |
|  | | ≥2 | | | | | 1.16 (0.96-1.39) | | | | | | |  | | | |  |  |  |  |
| Insurance Status | | Uninsured | | | | | REF | | | | | | | 0.001 | | | |  |  |  |  |
|  | | Private Insurance | | | | | 0.79 (0.62-1.01) | | | | | | |  | | | |  |  |  |  |
|  | | Medicaid | | | | | 0.73 (0.58-0.92) | | | | | | |  | | | |  |  |  |  |
|  | | Medicare | | | | | 0.90 (0.68-1.19) | | | | | | |  | | | |  |  |  |  |
| Median Income | | ≤$47,999 | | | | | REF | | | | | | | 0.003 | | | |  |  |  |  |
|  | | $48,000-$62,999 | | | | | 0.83 (0.73-0.95) | | | | | | |  | | | |  |  |  |  |
|  | | ≥$63,000 | | | | | 0.84 (0.72-0.98) | | | | | | |  | | | |  |  |  |  |
| **Tumor Factors** |  | | | |  |  | | | |  |  | | | |  | | | | |  | |
| Tumor Grade | | Well | | | | | REF | | | | | | | <0.001 | | | |  |  |  |  |
|  | | Moderate | | | | | 1.20 (1.01 - 1.43) | | | | | | |  | | | |  |  |  |  |
|  | | Poor | | | | | 1.39 (1.16 - 1.67) | | | | | | |  | | | |  |  |  |  |
|  | | Anaplastic | | | | | 1.46 (1.14 - 1.87) | | | | | | |  | | | |  |  |  |  |
| AJCC Pathological T Classification | | T1 | | | | | REF | | | | | | | <0.001 | | | |  |  |  |  |
|  | | T2 | | | | | 0.95 (0.77-1.17) | | | | | | |  | | | |  |  |  |  |
|  | | T3 | | | | | 1.83 (1.51-2.23) | | | | | | |  | | | |  |  |  |  |
|  | | T4 | | | | | 1.72 (1.41-2.11) | | | | | | |  | | | |  |  |  |  |
| AJCC Pathological N Stage | | N0 | | | | | REF | | | | | | | <0.001 | | | |  |  |  |  |
|  | | N1 | | | | | 1.61 (1.43-1.81) | | | | | | |  | | | |  |  |  |  |
|  | | N2 | | | | | 2.32 (2.00-2.69) | | | | | | |  | | | |  |  |  |  |
|  | | N3 | | | | | 2.30 (1.92-2.77) | | | | | | |  | | | |  |  |  |  |
| Lymphovascular Invasion | | Absent | | | | | REF | | | | | | | <0.001 | | | |  |  |  |  |
|  | | Present | | | | | 1.36 (1.18-1.55) | | | | | | |  | | | |  |  |  |  |
| **Treatment Factors** | | | |  | | | | |  | | | | | | |  | | | | |  |
| Adjuvant Radiotherapy | | No | | | | | REF | | | | | | | <0.001 | | | |  |  |  |  |
|  | | Yes | | | | | 0.87 (0.75-1.01) | | | | | | |  | | | |  |  |  |  |
| Adjuvant Chemotherapy * Margin Status | | R0 + noAC | | | | | REF | | | | | | | <0.001 | | | |  |  |  |  |
|  | | R0 + AC | | | | | 0.85 (0.77-0.94) | | | | | | |  | | | |  |  |  |  |
|  | | R1 + noAC | | | | | 2.00 (1.55-2.59) | | | | | | |  | | | |  |  |  |  |
|  | | R1 + AC | | | | | 0.69 (0.48-1.00) | | | | | | |  | | | |  |  |  |  |

** Abbreviations: AC: adjuvant chemotherapy, AJCC: American Joint Commission on Cancer, CDCC: Charlson-Deyo comorbidity, CI: confidence interval, REF: referent **Additional variables included into the propensity matching omitted from tables were hospital factors (hospital distance), patient factors (race, education level, residence) and tumor factors (lymph nodes examined).*

Supplementary Table 5 Multivariable Cox regression model of survival of patients with resected ampullary adenocarcinoma in matched cohort, with interactions between chemotherapy and radiotherapy

|  | | |  | | | | | **Hazard ratio (CI_95%_)** | | | | | | **p-value** | |  |  |  |  |
| --- | --- | --- | --- | --- | --- | --- | --- | --- | --- | --- | --- | --- | --- | --- | --- | --- | --- | --- | --- |
| **Hospital Factors** | |  | | | | |  | | | | |  | | | |  |  |  |  |
| Center Volume | | | 1 (Lowest) | | | | | REF | | | | | | <0.001 | |  |  |  |  |
|  | | | 2 | | | | | 0.84 (0.72-0.98) | | | | | |  | |  |  |  |  |
|  | | | 3 | | | | | 0.94 (0.80-1.11) | | | | | |  | |  |  |  |  |
|  | | | 4 | | | | | 0.88 (0.74-1.05) | | | | | |  | |  |  |  |  |
|  | | | 5 (Highest) | | | | | 0.82 (0.68-0.98) | | | | | |  | |  |  |  |  |
| Facility Type | | | Community | | | | | REF | | | | | | 0.2 | |  |  |  |  |
|  | | | Academic | | | | | 1.03 (0.91-1.16) | | | | | |  | |  |  |  |  |
|  | | | Others | | | | | 1.11 (0.96-1.29) | | | | | |  | |  |  |  |  |
| Facility Location | | | Northeast | | | | | REF | | | | | | 0.004 | |  |  |  |  |
|  | | | South | | | | | 1.19 (1.05-1.35) | | | | | |  | |  |  |  |  |
|  | | | Midwest | | | | | 1.13 (0.99-1.29) | | | | | |  | |  |  |  |  |
|  | | | West | | | | | 1.20 (1.04-1.38) | | | | | |  | |  |  |  |  |
| **Patient Factors** | | |  | | | | |  | | | | | |  | | | |  |  |
| Year of Diagnosis | | | 2006-2007 | | | | | REF | | | | | | 0.1 | |  |  |  |  |
|  | | | 2008-2009 | | | | | 1.02 (0.87-1.20) | | | | | |  | |  |  |  |  |
|  | | | 2010-2011 | | | | | 0.72 (0.60-0.87) | | | | | |  | |  |  |  |  |
|  | | | 2012-2013 | | | | | 0.66 (0.55-0.79) | | | | | |  | |  |  |  |  |
|  | | | 2014-2016 | | | | | 0.68 (0.56-0.83) | | | | | |  | |  |  |  |  |
| Age at Diagnosis, years | | | 36-50 | | | | | REF | | | | | | <0.001 | |  |  |  |  |
|  | | | 51-65 | | | | | 1.49 (1.23-1.80) | | | | | |  | |  |  |  |  |
|  | | | 66-80 | | | | | 1.68 (1.36-2.08) | | | | | |  | |  |  |  |  |
|  | | | ≥80 | | | | | 2.60 (2.00-3.38) | | | | | |  | |  |  |  |  |
|  | | | Missing | | | | | 0.95 (0.23-3.92) | | | | | |  | |  |  |  |  |
| Sex | | | Male | | | | | REF | | | | | | <0.001 | |  |  |  |  |
|  | | | Female | | | | | 0.84 (0.77-0.92) | | | | | |  | |  |  |  |  |
| CDCC Score | | | 0-1 | | | | | REF | | | | | | <0.001 | |  |  |  |  |
|  | | | ≥2 | | | | | 1.17 (0.98-1.41) | | | | | |  | |  |  |  |  |
| Insurance Status | | | Uninsured | | | | | REF | | | | | | 0.1 | |  |  |  |  |
|  | | | Private Insurance | | | | | 0.88 (0.72-1.07) | | | | | |  | |  |  |  |  |
|  | | | Medicaid | | | | | 1.09 (0.84-1.41) | | | | | |  | |  |  |  |  |
|  | | | Medicare | | | | | 0.94 (0.77-1.15) | | | | | |  | |  |  |  |  |
| Median Income | | | ≤$47,999 | | | | | REF | | | | | | 0.005 | |  |  |  |  |
|  | | | $48,000-$62,999 | | | | | 0.83 (0.73-0.94) | | | | | |  | |  |  |  |  |
|  | | | ≥$63,000 | | | | | 0.84 (0.72-0.98) | | | | | |  | |  |  |  |  |
| **Tumor Factors** |  | | | |  |  | | | |  |  | |  | | | |  | |  |
| Tumor Grade | | | Well | | | | | REF | | | | | | <0.001 | |  |  |  |  |
|  | | | Moderate | | | | | 1.20 (1.01-1.43) | | | | | |  | |  |  |  |  |
|  | | | Poor | | | | | 1.39 (1.16-1.67) | | | | | |  | |  |  |  |  |
|  | | | Anaplastic | | | | | 1.46 (1.14-1.88) | | | | | |  | |  |  |  |  |
| AJCC Pathological T Classification | | | T1 | | | | | REF | | | | | | <0.001 | |  |  |  |  |
|  | | | T2 | | | | | 0.95 (0.77-1.17) | | | | | |  | |  |  |  |  |
|  | | | T3 | | | | | 1.82 (1.50-2.22) | | | | | |  | |  |  |  |  |
|  | | | T4 | | | | | 1.72 (1.40-2.10) | | | | | |  | |  |  |  |  |
| AJCC Pathological N Stage | | | N0 | | | | | REF | | | | | | <0.001 | |  |  |  |  |
|  | | | N1 | | | | | 1.60 (1.43-1.80) | | | | | |  | |  |  |  |  |
|  | | | N2 | | | | | 2.32 (2.00-2.69) | | | | | |  | |  |  |  |  |
|  | | | N3 | | | | | 2.30 (1.91-2.76) | | | | | |  | |  |  |  |  |
| Margin Status | | | Negative | | | | | REF | | | | | | <0.001 | |  |  |  |  |
|  | | | Positive | | | | | 1.65 (1.37-1.99) | | | | | |  | |  |  |  |  |
| Lymphovascular Invasion | | | Absent | | | | | REF | | | | | | <0.001 | |  |  |  |  |
|  | | | Present | | | | | 1.35 (1.18-1.55) | | | | | |  | |  |  |  |  |
| **Treatment Factors** | | | |  | | | | |  | | | | | |  | | | | |
| Adjuvant Chemotherapy * Adjuvant Radiotherapy | | | NoAC + No ART | | | | | REF | | | | | | <0.001 | |  |  |  |  |
|  | | | NoAC + ART | | | | | 0.81 (0.68-0.96) | | | | | |  | |  |  |  |  |
|  | | | AC + No AT | | | | | 0.65 (0.59-0.72) | | | | | |  | |  |  |  |  |
|  | | | AC + ART | | | | | 0.56 (0.47-0.66) | | | | | |  | |  |  |  |  |

**Abbreviations: AC: adjuvant chemotherapy, AJCC: American Joint Commission on Cancer, ART: Adjuvant radiotherapy, CDCC: Charlson-Deyo comorbidity, CI: confidence interval, noAC: No adjuvant chemotherapy, REF: referent **Additional variables included into the propensity matching omitted from tables were hospital factors (hospital distance), patient factors (race, education level, residence) and tumor factors (lymph nodes examined).*

Supplementary Figure 1 Variation in receipt of adjuvant chemotherapy by center following resection for ampullary adenocarcinoma

Supplementary Figure 2 Overall survival of adjuvant chemotherapy following resection for ampullary adenocarcinoma stratified by nodal status in matched cohorts (A) N0 (B) N2

**A**

**B**

p=0.1

p=0.5

Supplementary Figure 3 Overall survival of adjuvant chemotherapy following resection for ampullary adenocarcinoma stratified by receipt of radiotherapy status in matched cohorts (A) No adjuvant radiotherapy (B) Adjuvant radiotherapy

**A**

**B**

p=0.2

p<0.001
